# Antimicrobial resistance markers distribution in *Staphylococcus aureus* from Nsukka, Nigeria

**DOI:** 10.1186/s12879-024-09126-1

**Published:** 2024-03-15

**Authors:** Martina C. Agbo, Ifeoma M. Ezeonu, Beatrice O. Onodagu, Chukwuemeka C. Ezeh, Chizoba A. Ozioko, Stephen C. Emencheta

**Affiliations:** 1 Department of Pharmaceutical Microbiology and Biotechnology, University of Nigeria, Nsukka, Nigeria; 2 Department of Microbiology, University of Nigeria, Nsukka, Nigeria; 3 Microbiology Laboratory Unit, University of Nigeria Teaching Hospital, Enugu, Nigeria; 4 VBlab-Laboratory of Bacterial Viruses, University of Sorocaba, 18023-000 Sorocaba, SP Brazil

**Keywords:** *S. Aureus*, Antibiotic resistance, Genes, Markers

## Abstract

**Background:**

Multidrug resistance in *Staphylococcus aureus* continues to influence treatment complications in clinical settings globally. Multidrug-resistant-*S. aureus* (MDR-SA) is often genetically driven by resistance markers transferable in pathogenic strains. This study aimed to determine the distribution of resistance markers in clinical isolates of *S. aureus* in Nsukka, Nigeria.

**Methods:**

A total of 154 clinical samples were cultured on mannitol salt agar. Isolates were characterized using conventional cultural techniques and confirmed by PCR detection of *S. aureus*-specific *nuc* gene. Antibiotic resistance profiles of the isolates were determined against selected antibiotics using the disk-diffusion method, while screening for antibiotic resistance genes (*Mec A, Erm A, Erm B, Erm C, Van A*, and *Van B*) was by PCR.

**Results:**

A total of 98 isolates were identified as *S. aureus* by conventional methods. Of these, 70 (71.43%) were confirmed by PCR. Phenotypically, the isolates exhibited high degrees of resistance to oxacillin (95.72%), erythromycin (81.63%), and ertapenem (78.57%) and 75.51% and 47.30% against methicillin and vancomycin, respectively. Multiple antibiotic resistance indexes of the isolates ranged from 0.3 to 1, and the most prevalent pattern of resistance was oxacillin-ertapenem-vancomycin-erythromycin-azithromycin-clarithromycin-ciprofloxacin- cefoxitin-amoxicillin-clavulanic acid. PCR screening confirmed the existence of various antibiotic resistance makers among the strains, with the most common resistance genes found in the isolates being *Mec A* (32.14%), *Van A* (21.43%), *Van B* (10.71%), *Erm B* (10.71%), and *Erm C* (17.86%). None possessed the *Erm A* gene.

**Conclusion:**

The study supports the need for necessary action, including rational drug use, continuous surveillance, and deployment of adequate preventive and curative policies and actions.

**Supplementary Information:**

The online version contains supplementary material available at 10.1186/s12879-024-09126-1.

## Background

Antibiotic resistance continues to present global and public healthcare challenges. Increasingly pathogens are becoming resistant to commonly used antibiotics [1]. The African continent, particularly the West African countries, suffers the worst burden, with an estimated 27.3 deaths per 100,000 directly related to antibiotic resistance, in the region, described by the Financial Times [2] as “a pandemic that is already here” and attributable to specific multifactorial issues, ranging from poor diagnostic methods, prescribing habits, non-compliance to therapy, distribution of sub-standard or fake drugs, to inadequate surveillance [2–4]. However, the World Health Organization (WHO) has called for a multisectoral approach to combating the menace, with surveillance and reporting vital aspects of the action plan [1].

The antibiotic resistance prevalence in pathogens has increased in recent decades [5]. *S. aureus* is one of the most prevalent bacteria encountered in clinical settings and is a common cause of nosocomial and community-acquired infections, with almost 30% of the human population asymptomatically colonized with commensal strains [6, 7]. The bacterium is also heavily implicated in skin and soft tissue diseases, pneumonia, meningitis, wound infections, sepsis, abscess formation, osteomyelitis, endocarditis, food poisoning, and toxic shock syndrome (TSST) [8]. However, implicated disease management has become more difficult owing to the emergence of multidrug-resistant strains [1, 9]. In perspective, a systematic review by Tadesse et al. [4] of the status of antibiotic resistance in different parts of the world showed that methicillin-resistant *S. aureus* (MRSA) was reported in 7/79 (8.9%) of the considered studies. The study, however, also suggested that the reported rate could be an underestimation since cefoxitin is one of the most currently used antibiotics for MRSA sreening in many laboratories [4]. The other reports include erythromycin (33.9%), oxacillin (40.7%), and the least vancomycin resistance in the studies. Although comprehensive, the review highlighted the gap in aggregating studies from the African region. This could be attributed to having little information sourced from the area. They submitted Africa and Southeast Asia as regions without established antimicrobial microbial resistance (AMR) surveillance systems [4].

Continuous monitoring of resistant strains is essential to understand and potentially predict trends in antibiotic resistance patterns and establish an adequate infection control program, which invariably would inform clinical practice [3]. The knowledge of pathogens’ antimicrobial resistance patterns is essential to guiding empirical and pathogen-specific therapy [1]. There are, however, reports of false negative and positive results in conventional antimicrobial tests, hence the increasing call for gene-based screening methods for antibiotic resistance [10].

Generally, in Nigeria, antibiotic susceptibility assays against *S. aureus* are done using disk-diffusion and culturing on certain selective media such as vancomycin and methicillin screening agars to detect vancomycin-resistant *S. aureus* (VRSA) and methicillin-resistant *S. aureus* (MRSA), respectively. Recent studies are, however, employing DNA- methods such as polymerase chain reaction (PCR) to determine the presence of resistance markers within isolates of *S. aureus* [10]. The technique potentially enhances the specificity and accuracy in diagnosing strains and genotypic forms of multidrug-resistant strains from different hosts and environments [11, 12]. Currently, little or no information is available on the distribution of antibiotic resistance genes in *S. aureus* from Nsukka, a Southeastern town in Nigeria. Thus, this study aims to determine the phenotypic and genotypic (*Mec A, Erm A, B, C*, and *Van A* and *B*) antibiotic resistance (AR) pattern among clinical isolates of *S. aureus* in Nsukka, Southeastern, Nigeria.

## Methods

### Specimen collection and bacterial identification

A total of one hundred and fifty-four (154) clinical samples were collected from fifteen (15) hospitals in and around Nsukka, Southeastern Nigeria, between March and July 2021. Of the 154 clinical samples, 49 (36.84%), 103 (31.58%), and 3 (21.05%) were from wound/pus, urine, and ear swabs, respectively. The study was done per the Declaration of Helsinki [13], and appropriate approval (FPSRE/UNN/20/0008) was sought from the Research Ethics Committee of the Faculty of Pharmaceutical Sciences, University of Nigeria, Nsukka. Informed consent was also obtained from the patients before sample collection. Wound/pus swabs and ear swabs were inoculated into the sterile nutrient broth (Oxoid, UK) and incubated for 24 h at 37 ^o^C, after which a loopful of the broth culture of each sample was inoculated onto sterile mannitol salt agar (Oxoid, UK) and incubated at 37 ^o^C for 24 h. Urine samples were inoculated directly onto sterile mannitol salt agar plates. After incubation, colonies with yellowish pigments from the agar were characterized using standard microbiological techniques. The presumptive *S. aureus* isolates were subjected to the polymerase chain reaction (PCR) targeting the *S. aureus*-specific *nuc* gene [14].

### Antimicrobial susceptibility testing

The susceptibility of the clinical isolates to ten (10) antibiotics, including oxacillin (1 µg), cefoxitin (30 µg), amoxicillin-clavulanic (20/10) µg, ertapenem (30 µg), vancomycin (30 µg), amikacin (10 µg), erythromycin (15 µg), azithromycin 15 µg, clarithromycin 15 µg, and ciprofloxacin (5 µg) (Oxoid, UK), were evaluated by agar disk-diffusion method on Muller-Hinton agar plates, as recommended by Clinical and Laboratory Standard Institute (CLSI) [15].

### Phenotypic detection of MRSA and VRSA isolates

Using standard procedures as described by CLSI [16], the phenotypic detection of MRSA strains was done using oxacillin (1 µg) and cefoxitin (30 µg) disks, while those of VRSA were detected using both vancomycin disk (30 µg) and agar screening medium. Particularly, for vancomycin-agar-screening, plates were prepared in-house by adding six (6) µg/ml of vancomycin to brain heart infusion (BHI) agar (Oxoid, UK). The bacterial suspension was inoculated using a micropipette to spot a 10 µl drop (final concentration 10^6^ CFU/ml) on the surface of the BHI agar plate containing six (6) µg/ml vancomycin and incubated for 24 h in ambient air at 35 ^o^C. The presence of more than one colony of the strain or light film of growth indicates presumptive reduced susceptibility to vancomycin. The inhibition zones for the oxacillin disk with a diameter of ≤ 10 mm for *S aureus* were considered resistant, and the inhibition zone for cefoxitin with a diameter of ≥ 20 and ≤ 19 mm were considered susceptible and resistant, respectively [16]. The phenotypic cefoxitin- and oxacillin-resistant and vancomycin-resistant isolates were subjected to polymerase chain reaction to detect the presence of *mec A, Van A, Van B, erm A*, *B*, and *C* genes.

### Determination of minimum inhibitory concentration (MIC) of Vancomycin

The MIC values of vancomycin were determined by the micro broth dilution method. Vancomycin suspension was prepared by dissolving 256 mg of vancomycin powder in 10 ml of sterile distilled water to obtain 25.6 mg/ml, after which further dilution (1:10 ) was done twice to achieve 256 µg/ml. A two-fold dilution of the prepared vancomycin concentration was done in a 96-well plate. Fifty microlitres (50 µl) of double-strength Muller-Hinton broth, 50 µl of the antibiotic dilutions, and 5 µl of the bacteria suspensions adjusted to 0.5 MacFarland standard and diluted (1:20) were mixed and incubated at 37 °C for 18 h. MICs, ≤ 2 µg/ml was considered sensitive, 4–8 µg/ml as intermediate, and ≥ 16 µg/ml as vancomycin-resistant *S. aureus*.

### DNA extraction and polymerase chain reaction (PCR) for detection of *S. Aureus*-specific genes

DNA samples were extracted from the isolates according to the method described by Katvoravutthichai et al. [17].. The PCR for detecting *S. aureus*-specific and antibiotics-resistant genes was carried out using the New England Bio Lab one Taq 2X master mix with standard buffer. Amplification was carried out in a 25 µl total volume of PCR mixture containing 12.5 µl of 1X master mix (England Bio Lab) with standard buffer, 20 µM Tris-HCl, 1.8 mM MgCl_2_, 22 mM NH_4_Cl, 22 mM KCl, 0.2 mM dNTPs, 5% glycerol, 0.06% GEPAL CA-630, 0.05%, Tween 20, 25 units/ml Taq DNA polymerase, 0.5 µl of 10 µM each of primers (Inqaba, Biotech, South Africa) (Table 1), 3 µl of the extracted DNA, and 8.5 µl of sterile Nuclease free water (Norgen, Biotek Corop, Canada). The PCR amplification program for the primers used is shown in Table 1. The PCR was performed in a thermal cycler machine (BIBBY)–scientific Ltd, UK. The PCR products were resolved on 1.5% agarose gel, stained with ethidium bromide (0.5 µg/ml), and electrophoresis was carried out at 70 volts for 90 min and visualized under UV Tran illuminator (Upland, USA) as similarly described by [18]. A 100 bp DNA ladder (New England Bio labs) was used as the DNA molecular weight marker. The study used only negative control which was the PCR master mix (2x master mix with standard buffer, forward and reverse primers and nuclease-free water) without the DNA.

**Table 1:** Primer Sequences and PCR Conditions Used

Target genes	Primer Sequence ( 5^1^ − 3^1^)	PCR Conditions	Amplicon size (bp)	References
*S.aureus*- specific *nuc* gene	F:GCGATTGATGGTGATACGG TT R:AGCCAAGCCTTGACGAA CTAAAGC	Initial denaturation of 94 ^o^C for 5 min; 35 cycles of denaturation of 94 ^o^C for 30 s; annealing at 55 ^o^C for 30s; extension at 72 ^o^C for 1 min; and final extension at 72 ^o^C for 10 min.	270	[12]
*Mec A*	F:AGTTCTGCAGTACCGGAT TG R:AAAATCGATGGTAAGGT TCGC	Initial denaturation of 94 ^o^C for 5 min;40 cycles of denaturation of 94 ^o^C for 30 s; annealing at 55 ^o^C for 30 s; extension at 72 ^o^C for 60 s; and final extension at 72 ^o^C for 5 min.	533	[19]
*Van A*	F:GGCAAGTCAGGTGAAGA TG R:ATCAAGCGGTCAATCAG TTC	Initial denaturation of 94 ^o^C for 5 min; 40 cycles of denaturation at 94 ^o^C for 1 min; annealing at 55 ^o^C for 1 min; extension at 72 ^o^C for 2 min; and final extension at 72 ^o^C for 5 min.	713	[20]
*Van B*	F:GTGACAAACCGGAGGCG AGGA R:CCGCCATCCTCCTGCAAA AAA	Initial denaturation of 94 ^o^C for 10 min; 30 cycles of denaturation of 94 ^o^C for 30 s; annealing at 50 ^o^C for 45 min; extension at 72 ^o^C for 30 s and final extension at 72 ^o^C for 10 min	430	[21]
*Erm A*	F:TATCTTATCGTTGAGAAG GGATT R:CTACACTTGGCTTAGGAT GAAA	Initial denaturation of 94 ^o^C for 5 min; 30 cycles of denaturation of 95 ^o^C for 1 min; annealing at 55 ^o^C for 30 s; extension at 72 ^o^C `for 2 min and final extension at 72 ^o^C for 10 min	139	[22]
*Erm B*	F:CTATCTGATTGTTGAAGA AGGATT R:GTTTACTCTTGGTTTAGG ATGAAA		142	
*Erm C*	F:CTTGTTGATCACGATAAT TTCC R:ATCTTTTAGCAAACCCGT ATTC		190	

## Results

### Prevalence of *S. Aureus* from clinical sample

**Fig. 1:**
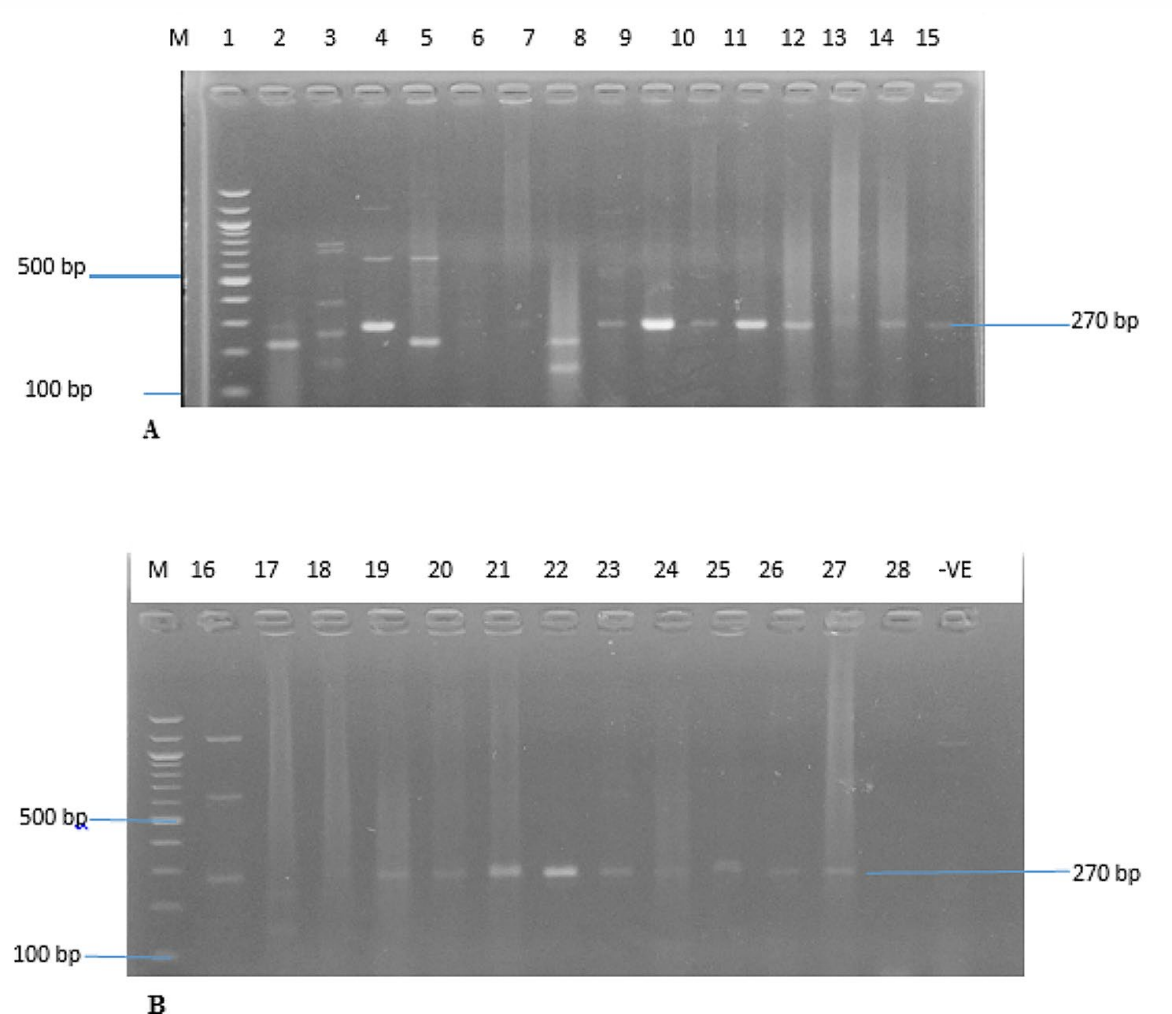
(**A** & **B**) are Amplified PCR products of *the nuc* gene at (270 bp). Lane M: 100 bp DNA ladder, lanes 1 to 14 and 16 to 27 positive to *staphylococcus aureus*

A total of ninety-eight (98) presumptive isolates of *S. aureus* were obtained from one hundred and fifty-four examined (154) clinical samples. A total of seventy (70) (71.43%) of the ninety-eight (98) isolates were confirmed as *S. aureus* using the *S. aureus* specific-*nu*c gene (Fig. 1). Thus, the remaining 28 (28.57%) isolates showed no presence of the *S. aureus* specific-*nu*c gene.

### Antimicrobial susceptibility and phenotypic identification of Methicillin and Vancomycin-resistant *S. Aureus*

Antimicrobial susceptibility pattern revealed that most *S. aureus* isolates were resistant to oxacillin (95.92%), erythromycin (81.63%), ertapenem (78.57%), cefoxitin (79.50%), and vancomycin (35.71%). 81.63% was, however, sensitive to amikacin 81.63% (Table 2). The vancomycin-resistant isolates were observed to have minimum inhibitory concentrations (MIC) values ranging from 2 to ≥ 16 µg/ml. Seventy-four (74) (75.51%) of the presumptive *S. aureus* were detected as methicillin-resistant phenotypically using cefoxitin (FOX) and oxacillin (OXA) disks. Thirty-five (35) (47.30%) of the methicillin-resistant *S. aureus* (MRSA) were detected as vancomycin-resistant *S. aureus* (VRSA) based on the antibiotic screening, using the presence/absence of growth the isolates on brain heart infusion agar supplemented with six (6) µg/ml of vancomycin. Most (96.94%, *n* = 95) of the *S. aureus* strains were multidrug-resistant (resistant to three or more antibiotics of at least two classes) with multiple antibiotic resistance (MAR) index greater than 0.2. A total of ninety-five different resistant patterns were obtained, with fourteen (14) strains exhibiting resistance to nine (9) (OX-EPT-VA-E-AZM-CLR-CIP-FOX-AMC) of the ten (10) antimicrobials tested. In all the isolates resistant to vancomycin (*n* = 35), at least five (5) other antibiotics were considered ineffective for their in vitro inhibition (Table 3).

### Genotypic characterization of resistance genes

**Fig. 2:**
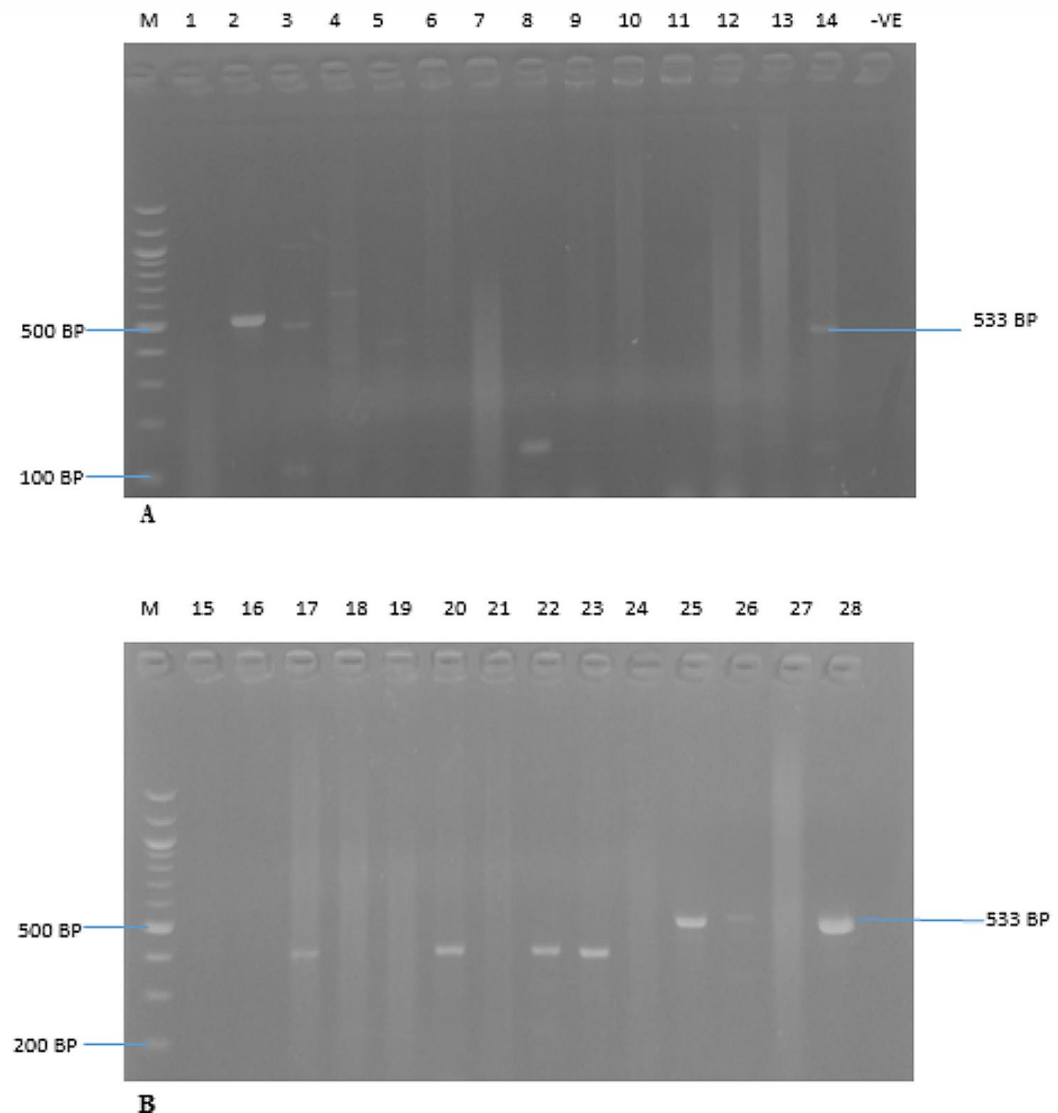
(**A** & **B**) are Amplified PCR products of the *mecA* gene at (533 bp). Lane m: 100 bp ladder. Lanes 2, 3, 14, 25, 26 and 28 positive to *MecA* gene

**Fig. 3:**
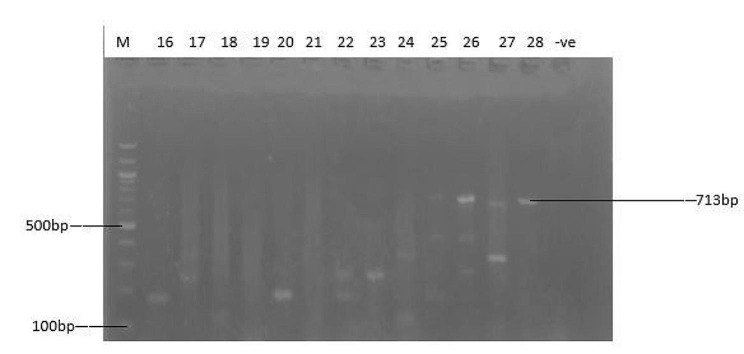
Amplified PCR Products of *Van A* gene at (713 bp); lane M: 100 bp ladder. Lane 25 to 28 positive to *Van A* gene for vancomycin-resistant *S. aureus* (VRSA) isolates

**Fig. 4:**
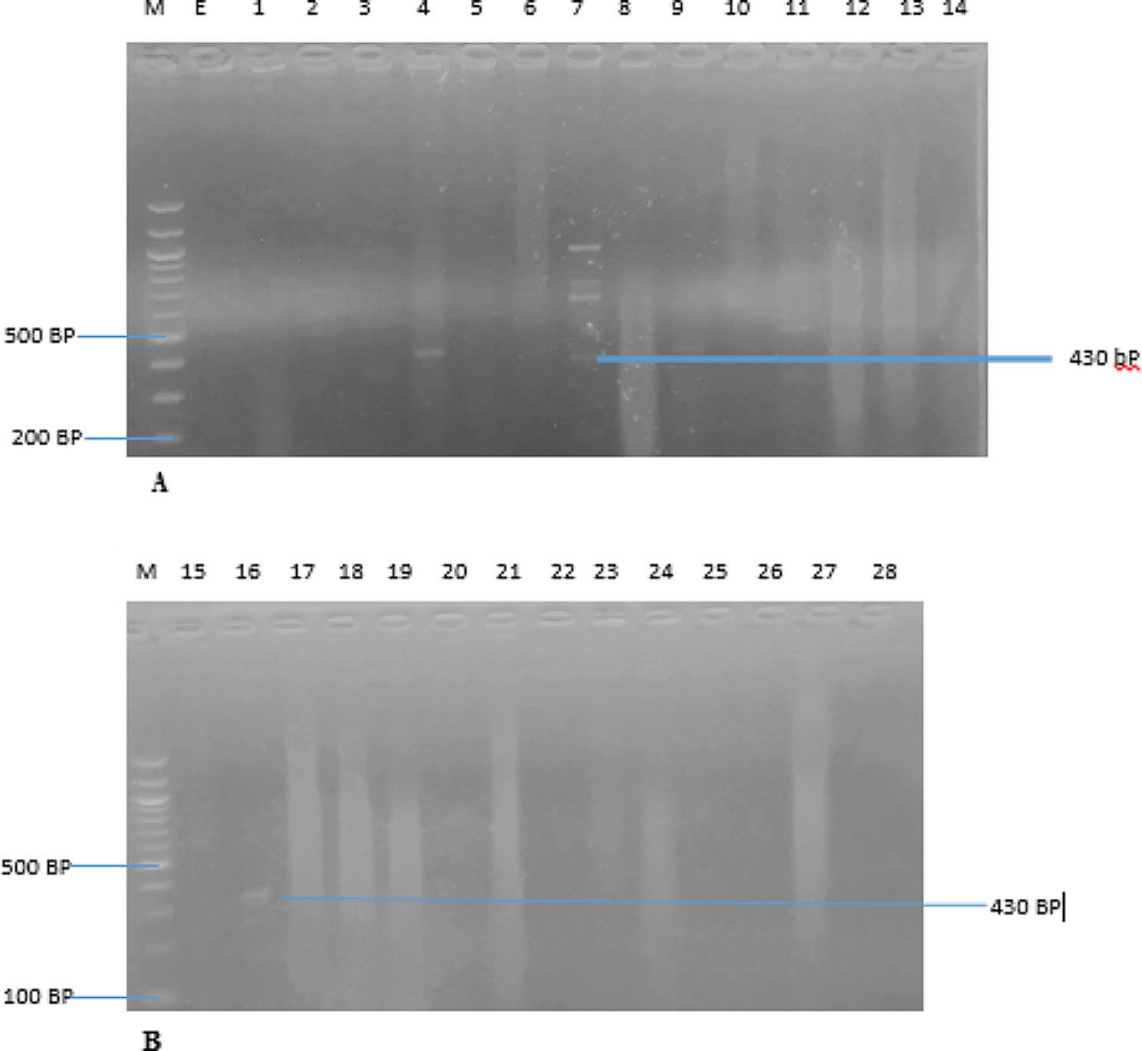
(**A** & **B**) are Amplified PCR products of the *Van B* gene at (430 bp). Lane M: 100 bp ladder; lanes 4, 7, and 16 positives to *Van B* gene for vancomycin-resistant *S. aureus* (VRSA) isolates

**Fig. 5:**
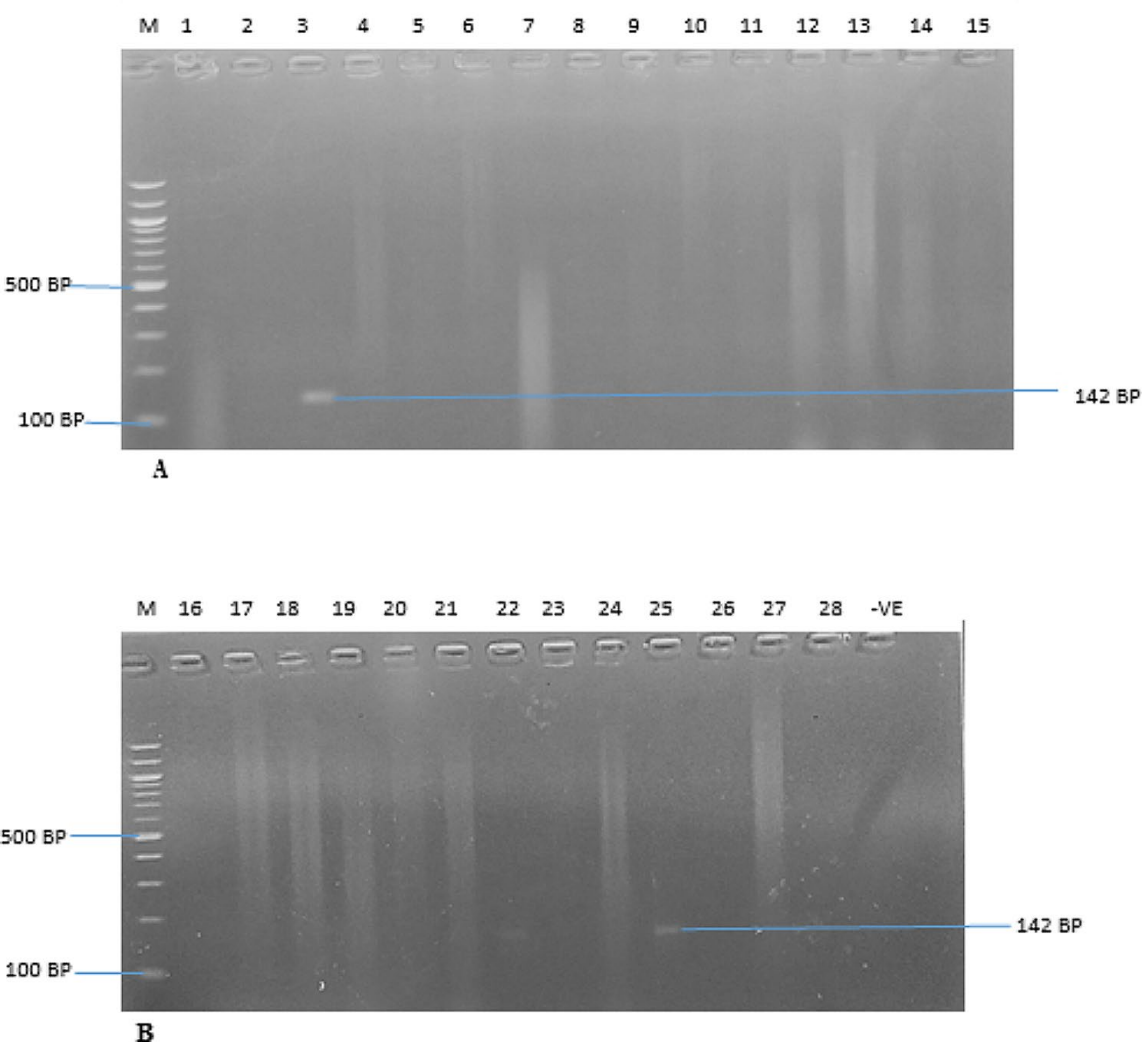
(**A** & **B**) are Amplified PCR products of *erm B* at (142 bp). Lane M: 100 bp ladder; Lane 3, 22, and 25 positives to *erm B* gene for Erythromycin resistant *S. aureus*

**Fig. 6:**
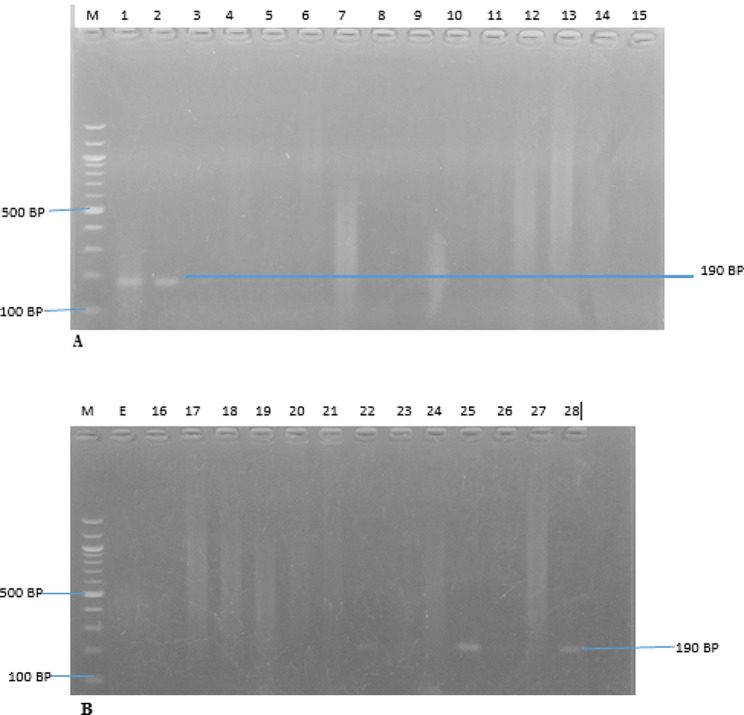
(**A** & **B**) are Amplified PCR products of *Erm C* at (190 bp). Lane M: 100 bp ladder lane. Lane 1, 2, 22, 25 and 28 positive to *Erm C* gene for Erythromycin resistant *S. aureus*

The identification of MRSA, VRSA, and the erythromycin-resistant strains were further confirmed using polymerase chain reaction (PCR), with the detection of the presence of *Mec A*, *Van A*, and *B*, and *Erm A, B* and *C* genes. The *Mec A*, *Van A*, *Van B*, and *Erm A, B*, & *C* genes were amplified from all the cefoxitin, oxacillin, macrolides and vancomycin-resistant isolates, respectively. The PCR results revealed that nine (9) *S. aureus* isolates (32.14%; 9/28) harbour the *Mec A* gene (Fig. 2), while six (6) (21.43%; 6/28) and four (4) (14.29%; 4/28) of the vancomycin-resistant isolates carried *Van A* and *Van B* genes, respectively (Figs. 3 and 4). The resistant isolates were also screened for three (3) genes, including *Erm A, B*, and *C*, as the primary markers of macrolide resistance. The positive isolates for the *Erm A, B, and C* genes showed 139, 142, and 190 bp bands, respectively (Figs. 5 and 6). The *Erm B* and *C* genes were present in three (3) (10.71%; 3/28) and five (5) isolates (17.86; 5/28) of erythromycin-resistant isolates, respectively, while *Erm A* was however absent.

### Distribution of the resistance markers

Based on the distribution of antimicrobial resistance genes, 26.67% of the isolates harboured three (3) of the resistance makers (*Van A, Mec A* and *Erm B Or Erm C*), while 6.67% of the isolates had *Van A, Mec A, Erm B* and *Erm C* gene combinations. (Table 4). However, the study did not detect antibiotic resistance determinant *Erm* A.

**Table 2:** The antibiotic susceptibility patterns of *S. aureus*

Antibiotics group	Antibiotic	Resistant No (%)	Susceptible No (%)
Penicillin	Oxacillin	94 (95.72)	4 (4.08)
	Cefoxitin	78 (78.59	20 (20.41)
Β-Lactamase inhibitors combination	Amoxicillin/clavulanic acid	70 (71.43)	28 (28.57)
Carbapenems	Ertapenem	77 (78.57)	19 (19.39)
Aminoglycosides	Amikacin	19 (19.39)	80 (81.63)
Macrolides	Azithromycin	75 (76.53)	23 (23.46)
	Clarithromycin	68 (69.39)	30 (30.61)
	Erythromycin	80 (81.63)	18 (18.37)
Fluoroquinolones	Ciprofloxacin	75 (76.53)	23 (23.47)
Glycopeptides	Vancomycin	35 (35.71)	63 (64.29)

**Table 3:** Antibiotic Resistance Patterns of the Multidrug-Resistant *S.aureus*

No. of Antibiotics resistant	Resistance patterns	Frequency of occurrence
10	OX-Ept-Va-Ak-E-Azm-CLR-Cip-Fox-Amc	4
9	OX-Ept-Va-E-Azm-CLR-Cip-Fox-Amc	14
	OX-Ept-Va- Ak-E-Azm-CLR-Cip-Fox-Amc	1
	OX-Ept- Ak-E-Azm-CLR-Cip-Fox-Amc	2
	OX-Va- Ak-E-Azm-CLR-Cip-Fox-Amc	1
8	OX-Ept-E- Azm-ClR-Cip-Fox-Amc	10
	OX-Va-E- Azm-ClR-Cip-Fox-Amc	1
	OX- Ept-Va-E- Azm-ClR-Cip-Fox-	1
	OX- Ept-Va-E- Azm-ClR-Fox-Amc	2
	OX- Ept-Va-E- Azm-ClR-Cip-Amc	1
	OX- Ept-Ak -E- Azm-ClR-Fox-Amc	2
	OX- Ept-Va -E- Azm-ClR-Fox-Amc	1
7	OX- Ept-E -Azm-ClR-Fox-Amc	3
	OX- Va-E -Azm-ClR- Cip-Fox	2
	OX-E -Azm-ClR- Cip-Fox-Amc	3
	OX-Ept-E- Azm-ClR- Cip-Fox-	3
	OX-Ept-E- Azm-ClR- Cip-Amc-	1
	OX-Ept- Va-E- Azm-ClR- Amc-	1
	OX-Ept -E- Azm-ClR- Fox-Amc-	2
6	OX-E- Azm-ClR-Cip- Fox	4
	OX-Va- Ak-E-Azm-ClR	1
	OX -Azm-ClR-Cip-Fox-Amc	1
	OX–Ept-E-Azm-ClR-Cip	2
	OX–Ept-E-Azm-ClR-Cip	1
	OX–Ept–Va-Cip-Fox-Amc	1
	OX–E-Azm-CLR-Cip-Amc	1
	OX-Ept-E-Azm-Cip-Amc	1
	Ox-Ept-Ak-Azm-Cip-Fox	1
	Ox-Ept-Ak-Cip-Fox-Amc	1
	Ox-Ept-E-Azm-CLR-Amc	1
5	OX-Ept-Cip-Fox-Amc	4
	OX-E-Azm-CLR-Cip	1
	OX-Ept-Cip-Fox-Amc	3
	Ox-Ept-Va-Fox-Amc	3
	OX-Ept-Va-E-Cip	1
	OX-Ept-E-ClR-Amc	1
	OX-Ept-E-azm-clR	1
	OX-Ept-E-Fox-Amc	1
4	0X-E-Azm-clR	6
	OX-Ept-E-Cip	1
	OX-E-Azm-clR	1
3	E-Azm-Cip	1
**Total**		**95 MDR**

Key: OX: Oxacillin; Va: Vancomycin; Ept: ertapenem; E: erythromycin; AZM: Azithromycin; Cip: ciprofloxacin; Fox: cefoxitin; Amc: Amoxicillin/clavulanic acid

**Table 4:** Distribution of antimicrobial resistance genes among the clinical isolates of *S. aureus*

Sample source	Phenotypic resistance	Genotypic resistance
Wound	OX, Ept, Va, E, Azm, ClR	*Erm C*
Wound	Ox, Ept, Va, E, Azm, Clr, Cip, Fox, Amc	*Van A, Mec A, Erm C*
Ear	OX, Ept, Va, E, Fox	*Van A, Mec A, Erm B*
Wound	OX,Ept,VaAk,E,Azm,CLR,Cip, Fox,Amc	*Van B*
Wound	Ox, Ept, Va, Ak, E, Azm, ClR, Cip, Fox, Amc	*Van B*
Wound	Ox, Ept, Va, Fox, Amc	*Van B*
Wound	Ox, Va, Ept, E, Azm, CLR, Fox, Amc	*Van B*
Wound	Ox, Ept, E, Azm, CLR	*MecA*
Urine	Ox, Ept, Va, E, Ak, Cip, Amc	*MecA*
Urine	Ox, Ept, Va, E, Azm, CLR, Cip, Fox, Amc	*Mec A, Erm B, Erm C*
Urine	Ox, E, Azm, CLR, Cip, Fox	*Mec A*
Urine	Ox, Ept, Va, E, Azm, CLR, Cip, Fox, Amc	*Van A, Mec A, Erm B, Erm C*
Urine	Ox, Ept, E, Azm, ClR, Cip, Fox, Amc	*Van A, Mec A*
Urine	Ox, Ept, Va, E, Azm, CLR, Cip, Fox, Amc	*Van A*
Urine	Ox, Va, E, Fox, Cip, Azm	*Van A, Mec A, Erm C*

Key: OX: Oxacillin; Va: Vancomycin; Ept: ertapenem; E: erythromycin; AZM: Azithromycin; Cip: ciprofloxacin; Fox: cefoxitin; Amc: Amoxicillin/clavulanic acid. *Van A, Van B* genes: encodes for Vancomycim, *Erm A, B, C* genes: encodes erythromycin ribosomal methylase ABC, *Mec A* gene: encodes for methicillin-resistance [11, 19]

## Discussion

The widespread use and misuse of antimicrobial drugs have led to a general rise in the emergence of resistant pathogens, especially the prevalent public health-implicated bacteria. For Africa, the problem is further compounded by other factors, including the proliferation of fake and counterfeit medicines, poor prescribing habits, non-compliance to prescribed treatments and the lack of established surveillance systems. This increase in the drug-resistant virulent strains has become a severe problem in treating and controlling staphylococcal infections. The susceptibility test profiles obtained in the present study showed that a significant percentage of the *S. aureus* isolates were resistant to most of the commonly used antibiotics. As already mentioned, ninety-eight (98) isolates were obtained from clinical samples, including ear swabs, wounds, and urine, with seventy (70) confirmed as *S. aureus* by PCR detection of the specific *nuc* gene which the remaining twenty-eight (28) lacked. All the isolates were screened against a panel of ten (10) antibiotics representing seven antibiotic classes. The highest levels of resistance were recorded with penicillins (Oxacillin and cefoxitin) and macrolides (erythromycin and azithromycin), while the lowest resistance was with the aminoglycosides (amikacin). This indicates that the aminoglycosides antibiotic class are more effective agent compared to the alternatives tested in the study. The high resistance rates observed in this study agree with other studies [23, 24]. A meta-analysis done by Deyno et al. [25] in another African country, Ethiopia, further highlighted and supported our finding, reporting the increasing resistance of disease-causing *S. aureus* against most antibiotics and most worryingly to vancomycin. Africa, being a developing continent should be wary as it poses a serious impeding consequence if unchecked.

The resistance level recorded against vancomycin is similar to some studies, including Maalik et al. [24], who showed high resistance rates of *S. aureus* to vancomycin in Kasur, Punjab, Pakistan. It calls for great concern, with vancomycin representing the standard therapy for invasive MRSA infections in humans. Other studies have also revealed the emergence of vancomycin-resistant strains in other parts of the world, including Egypt, with a resistance rate of 54.50% and Trahran, Iran [12, 14]. The increased frequency of resistance to this antibiotic entails the impending danger of hospital morbidity and possible mortality and suggests the need for combination therapy, adoption of alternatives, or inclusion of newer regimens for treating invasive staphylococcal infections. Moreover, proper campaigns are required to increase awareness among the general population about the impact of individual actions on developing resistance amongst the bacteria.

All the *S. aureus* isolates were multidrug-resistant, showing different multidrug resistance patterns to the ten (10) tested antimicrobials. The most common resistance pattern was OX-Ept-Va-E-Azm-CLR-Cip-Fox-Amc. The prevalence of MRSA phenotypically was 75.51%, which agrees with some other studies [26, 27]. This evolving trend and the potential rapid spread of resistance in *S. aureus* strains threaten disease management in animal and human health [28].

The phenotypic expression of antimicrobial resistance has been reported to be influenced by certain factors, including inoculum size, pH, salt concentration of medium, and incubation period [29]. Thus, detecting antibiotic resistance markers such as the *Mec A* gene by molecular methods, specifically PCR, is very helpful and valuable. The presence of the *Mec A* gene is considered the gold standard for defining MRSA, in addition to the new resistance genes *Mec C* and *Mec B*, which are homologues to *Mec A* [14, 30]. Here, PCR assays were used to screen for the presence of the *Mec A* gene among 74 presumptive MRSA strains. Of these phenotypic MRSA isolates, 32.14% expressed *Mec A* gene and, agreeing with the studies by Pournajaf et al. [31] and Alghizzi et al. [32], with both reporting similar occurrences of *Mec A* gene among the MRSA strains.

Erythromycin is a member of the macrolide family, which exhibits excellent potential in MRSA infections and is frequently used to treat staphylococcal skin and soft tissue infections (SSTIs) [33, 34]. Resistance to erythromycin is attributable to the presence of the *Erm* gene family encoding the methylase, which is responsible for the methylation of adenine in the 23 S rRNA ribosomal subunit. The isolates resistant to erythromycin phenotypically contained at least one erythromycin resistance gene. The distribution of resistance genes detected in this analysis demonstrates that *Erm C* (17.56%) was the predominant gene compared to *Erm B* (10.71%) and is in accordance with other studies [22, 23]. However, in countries such as Tunisia and Denmark, *Erm B* and *Erm A* genes were the most common clindamycin- and erythromycin-resistant genes, respectively [35]. The dissimilarities in the prevalence rate of MLSB resistance genes in different studies may be explained by the heterogeneous nature of erythromycin resistance or attributable to the loss of small plasmids carrying the *Erm* genes [35]. We also reported that 13.33% of the resistant *S. aureus* had both *Erm B* and *Erm C*, while none of the isolates harboured *Erm A*. Previous studies from different countries have also reported the simultaneous presence of two or more MLSB resistance genes [36].

With the emergence and spread of MRSA leading to treatment failures, vancomycin became the drug of choice against invasive MRSA infections [37]. However, increased and indiscriminate use of vancomycin over the years has led to the emergence of vancomycin resistance among *S. aureus* isolates. A vancomycin VRSA prevalence rate of 47.30% was recorded by phenotypic methods, with most having high MIC values (MIC 2 to ≥ 16 µg/ml) and is consistent with the study of Al-Amery et al. [12], who detected 27.60 and 54.59% of VRSA from dromedary camel and human. The high prevalence of these resistant strains is of particular public health concern. One of the mechanisms of vancomycin resistance in *S. aureus* is the transfer of plasmid containing Tn1546 and, thus, the *van A* gene cluster from vancomycin-resistant *Enterococcus spp* (VRE) [20]. This study screened the presence of *Van A* and *B* genes among the phenotypic vancomycin-resistant *S. aureus* using PCR, with some of the presumptive VRSA isolates harbouring both *Van A* and *Van B* genes and is similar to the study of Alghizzi et al., [32] and Karasin et al., [38] with the report of the presence of *Van A* and *Van B* resistance genes, respectively.

The distribution of resistance genes among *S. aureus* isolates demonstrates that *Mec A* was the predominant gene, followed by *Van A* gene and *Erm C*. Five (5) MRSA isolates carried *Van A, Erm B*, and *Erm C*. The coexistence of these genes in MRSA isolates was also observed in other studies [26, 39]. Additionally, the *Van A + Mec A + Erm B + Erm C* gene combination was detected in one (1) isolate. A low to moderate prevalence of antimicrobial resistance genes (ARGs) compared to the phenotypic resistance was also observed. Low prevalence of ARGs has been reported by Pekana et al. [40], Gao et al. [41], and Yang et al. [42], showing low expression of genotypic resistance in *S. aureus* in South Africa and China, respectively. Resistance mediated by other independent mechanisms, such as point mutations, biofilm formation, and antibiotic tolerance, could explain these findings [43].

## Conclusion

The study presented a high level of antibiotic resistance and also a significant distribution of resistance genes in the studied clinical *S. aureus* isolates. It raises the alarm of an impending antibiotic resistance crisis in the region. The study, however, recommends the rational drug use and adequate combination therapies, continuous bacterial resistance pattern surveillance, the establishment and deployment of efficient policies via constructive efforts of relevant stakeholders towards addressing the associated antibiotics resistance and their implications., and the advancement of research, including the development of rapid and reliable bacterial resistance screening techniques essential to instituting appropriate therapies/interventions.

### Electronic supplementary material

Below is the link to the electronic supplementary material.

Supplementary Material 1

## Data Availability

All queries regarding the study data could be addressed to the corresponding authors: Martina Chinonye Agbo, martina.agbo@unn.edu.ng, + 2348039705442; Stephen Chijioke Emencheta, stephen.emencheta@unn.edu.ng, + 2348140477129.

## Abbreviations

Amc: Amoxicillin/clavulanic acid
AR: Antibiotic resistance
ARGs: Antimicrobial resistance genes
AZM: Azithromycin
BHI: Brain heart infusion
Bp: Base pair
Cip: Ciprofloxacin
E: Erythromycin
Ept: Ertapenem
FOX: Cefoxitin
Fox: Cefoxitin
MDR: Multidrug resistance
MIC: Minimum inhibitory concentration
MRSA: Methicillin-resistant *S. aureus*
OX: Oxacillin
OXA: Oxacillin
PCR: Polymerase Chain Reaction
SSTIs: Staphylococcal skin and soft tissue infections
TSST: Toxic shock syndrome
Va: Vancomycin
VRE: Vancomycin-resistant *Enterococcus* spp
VRSA: Vancomycin-resistant *S. aureus*
WHO: World Health Organization
