# Supplementary material for: Antimicrobial resistance markers distribution in Staphylococcus aureus from Nsukka, Nigeria
Source: BMC Infect Dis. 2024 Mar 15;24:320. doi: 10.1186/s12879-024-09126-1 (PMC10943768; doi:10.1186/s12879-024-09126-1)
Supplement: Supplementary file 1 — Supplementary Material 1 [file 12879_2024_9126_MOESM1_ESM.docx]

**Supplementary files**


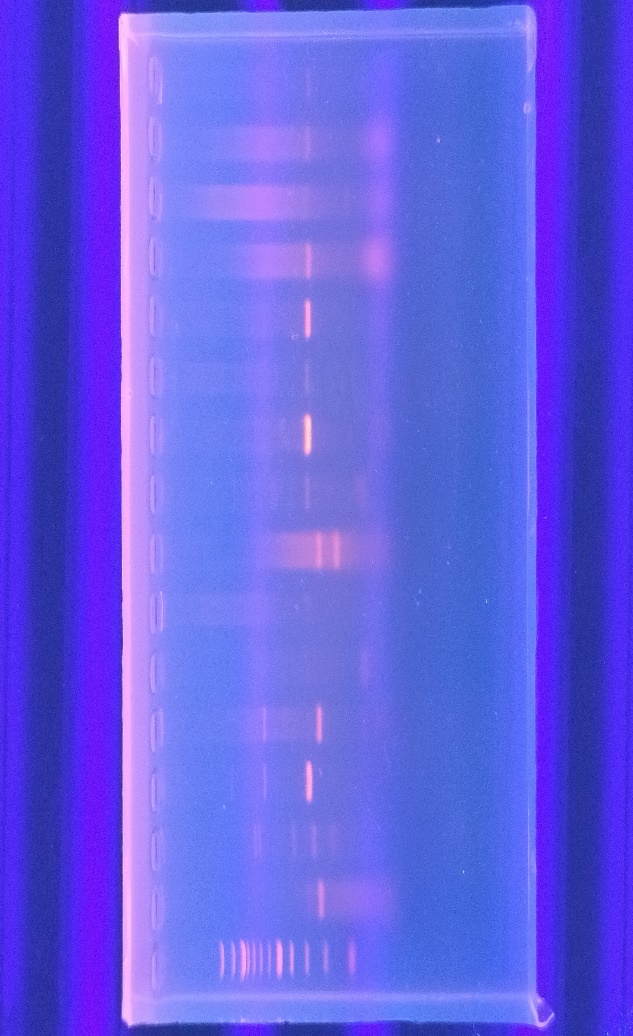


A


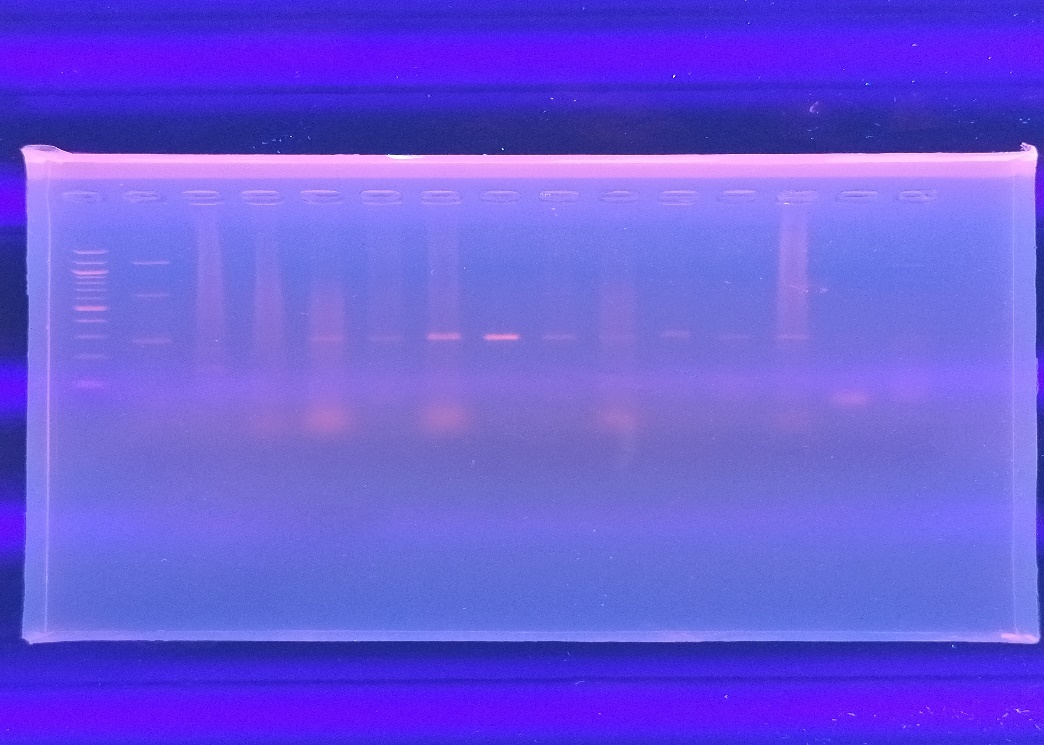


B

Figure 1: (A & B) are Amplified PCR products of *the nuc* gene at (270 bp). Lane M: 100 bp DNA ladder, lanes 1 to 14 and 16 to 27 positive to *staphylococcus aureus*


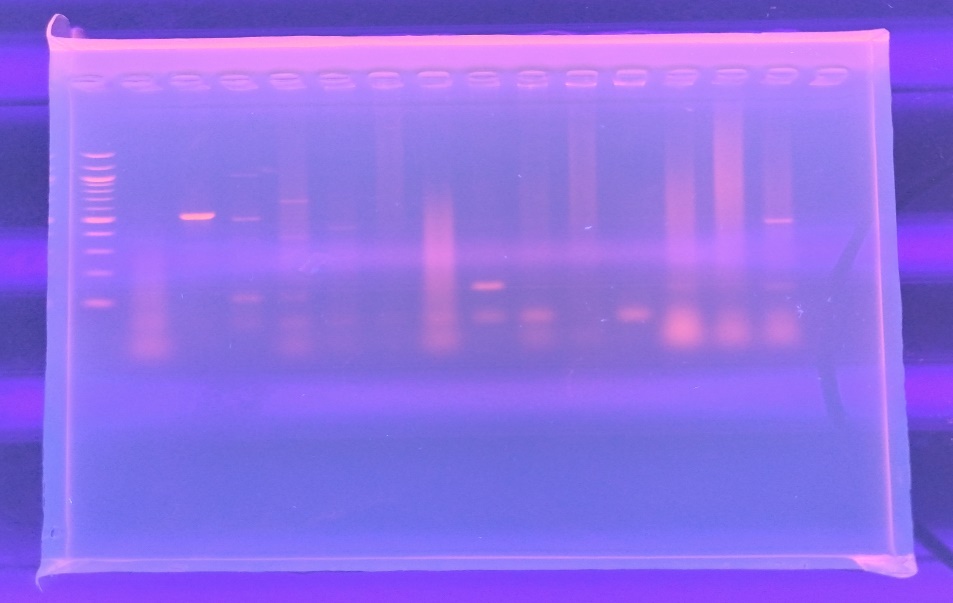


A


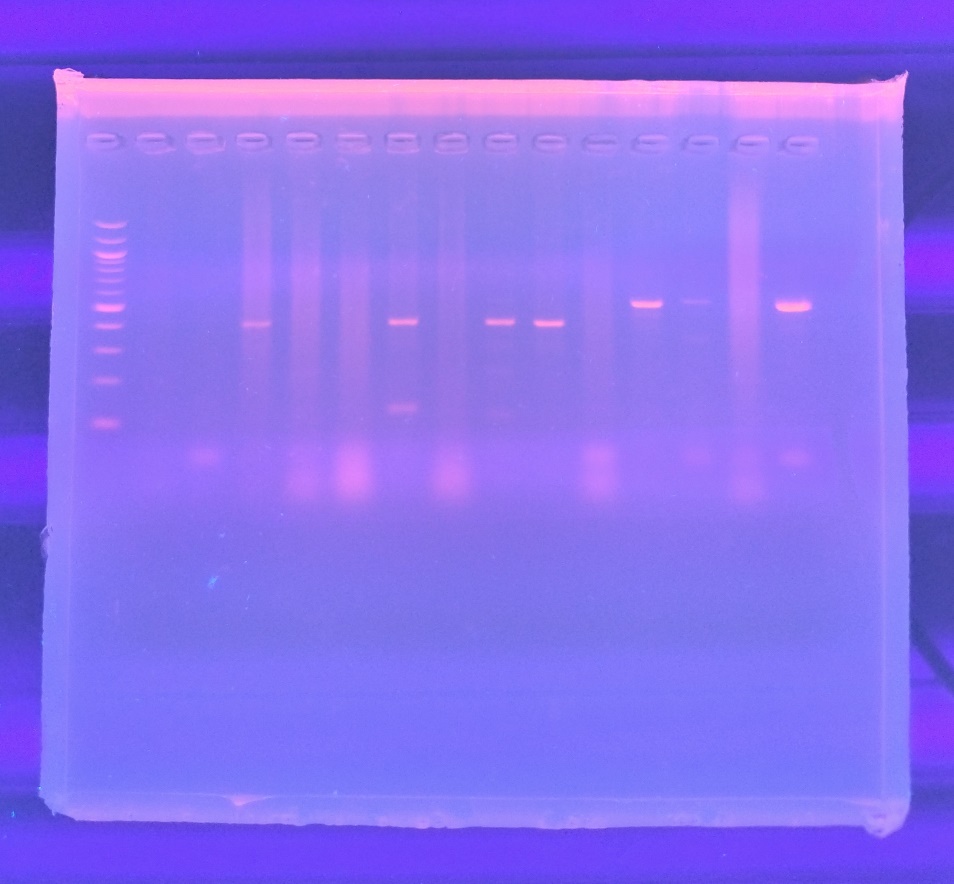


B

Figure 2: (A & B) are Amplified PCR products of the *mecA* gene at (533bp). Lane m: 100 bp ladder. Lane 2, 3, 14, 25, 26 and 28 positives to *MecA* gene.


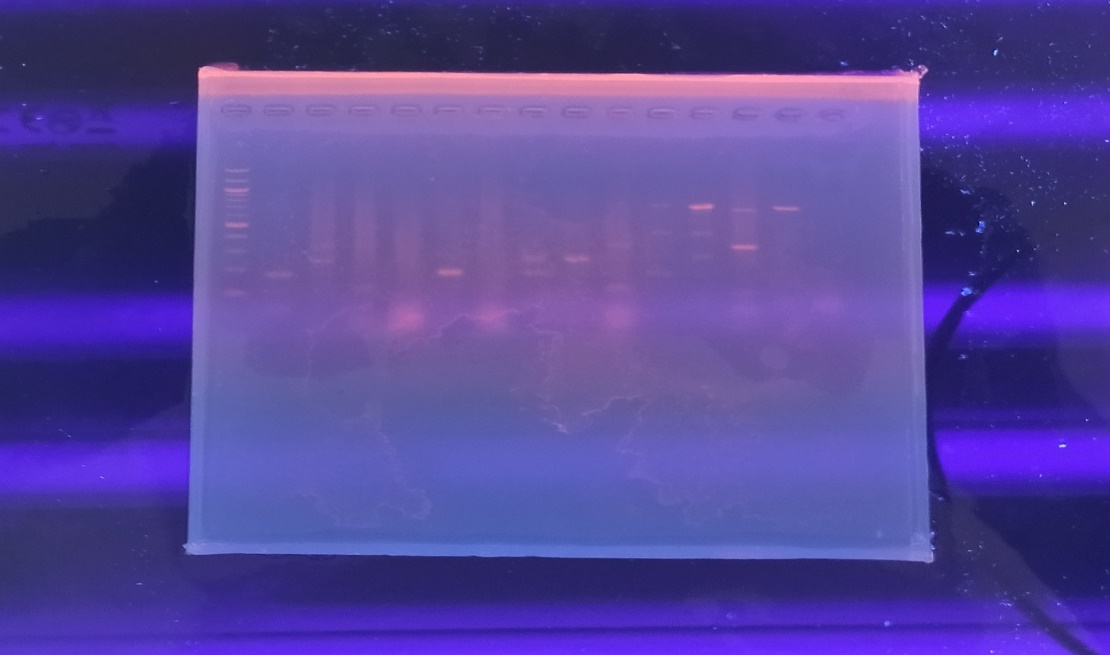


Figure 3: Amplified PCR Products of *Van A* gene at (713bp); lane M: 100bp ladder. Lane 25 to 28 positive to *Van A* gene for vancomycin-resistant *S. aureus* (VRSA) isolates.


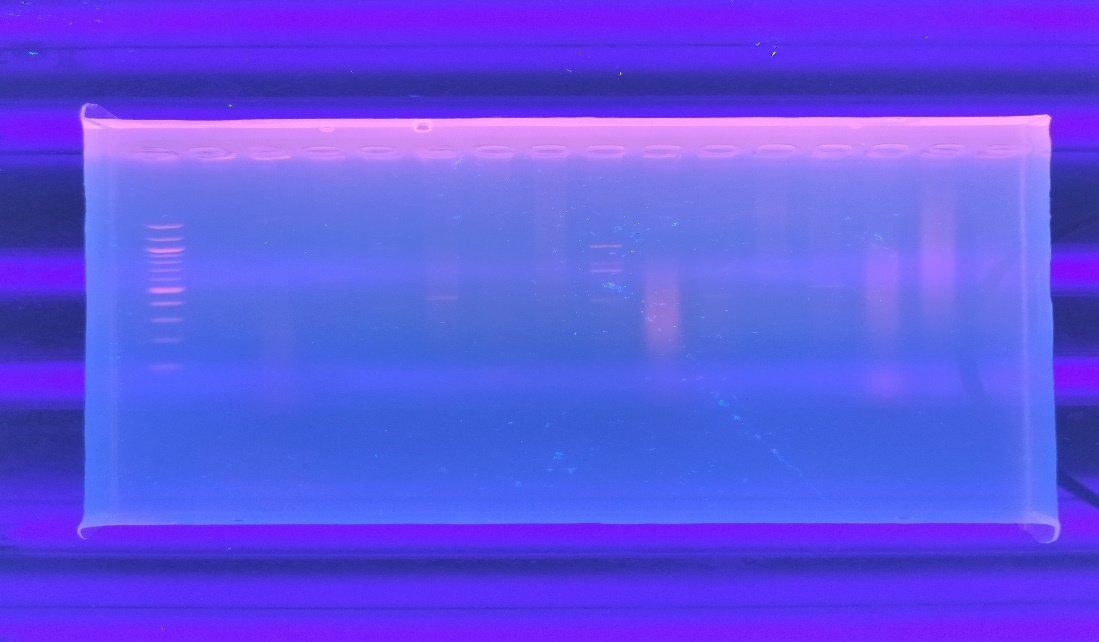


A


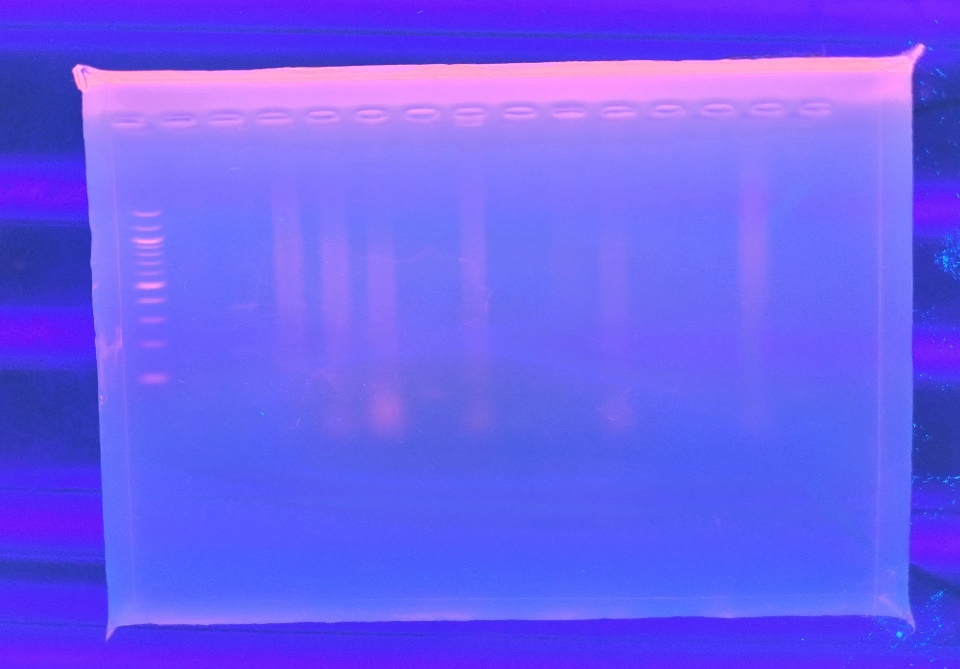


B

Figure 4: (A & B) are Amplified PCR products of the *Van B* gene at (430bp). Lane M: 100 bp ladder; lanes 4, 7, and 16 positives to *Van B* gene for vancomycin-resistant *S. aureus* (VRSA) isolates.


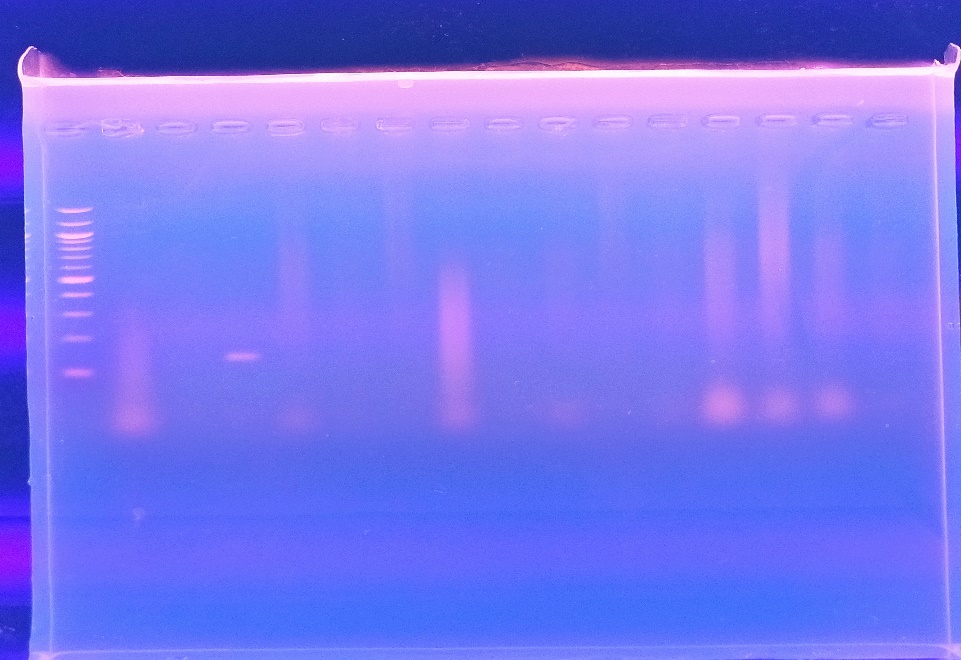


A


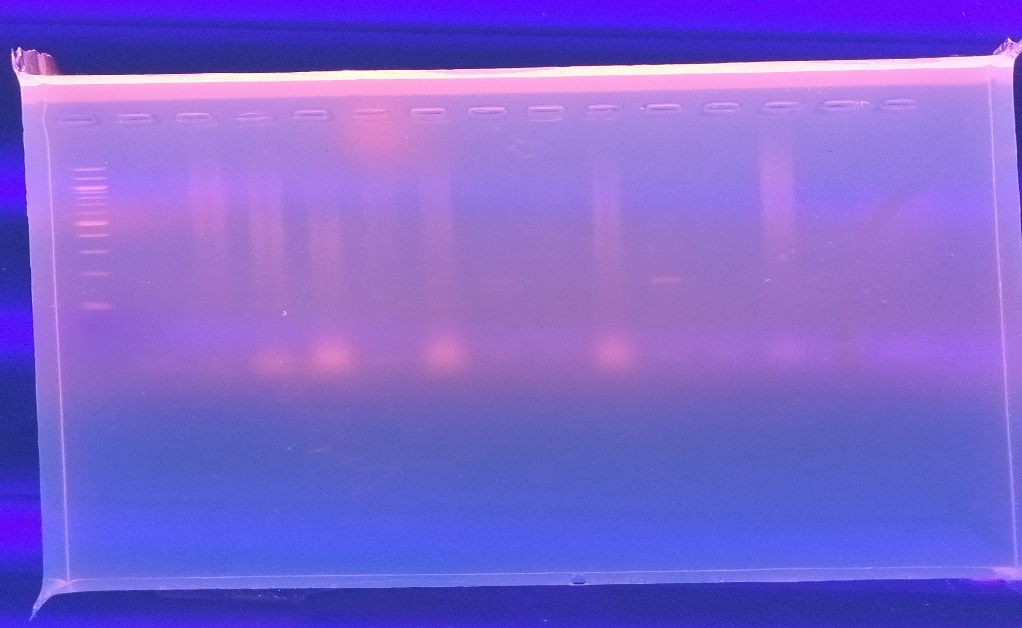


B

Figure 5: (A & B) are Amplified PCR products of *erm B* at (142 bp). Lane M: 100 bp ladder; Lane 3, 22, and 25 positives to *erm B* gene for Erythromycin resistant *S. aureus*.


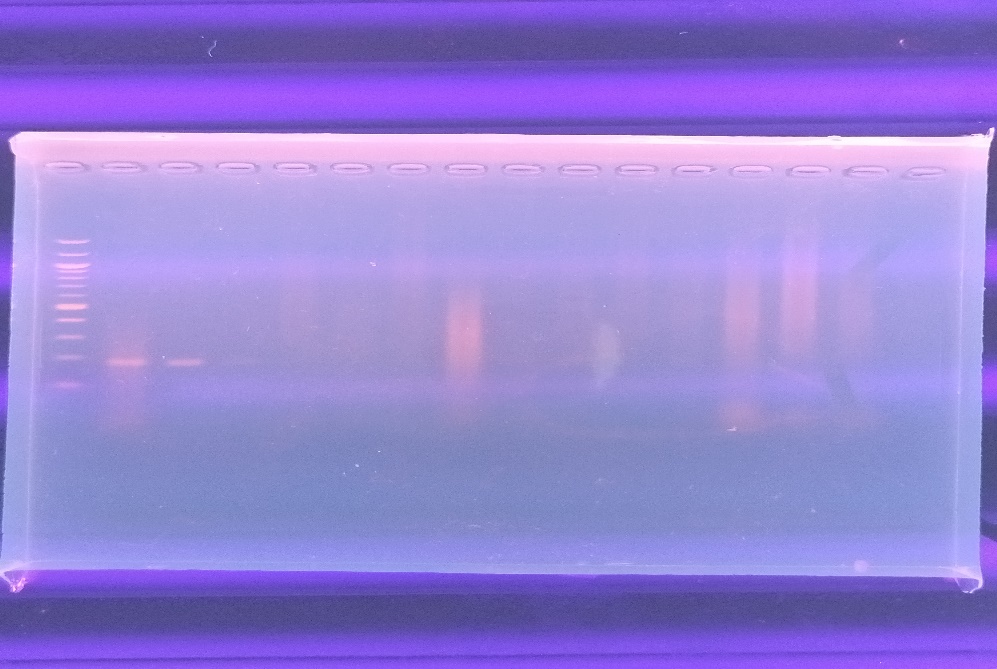


A


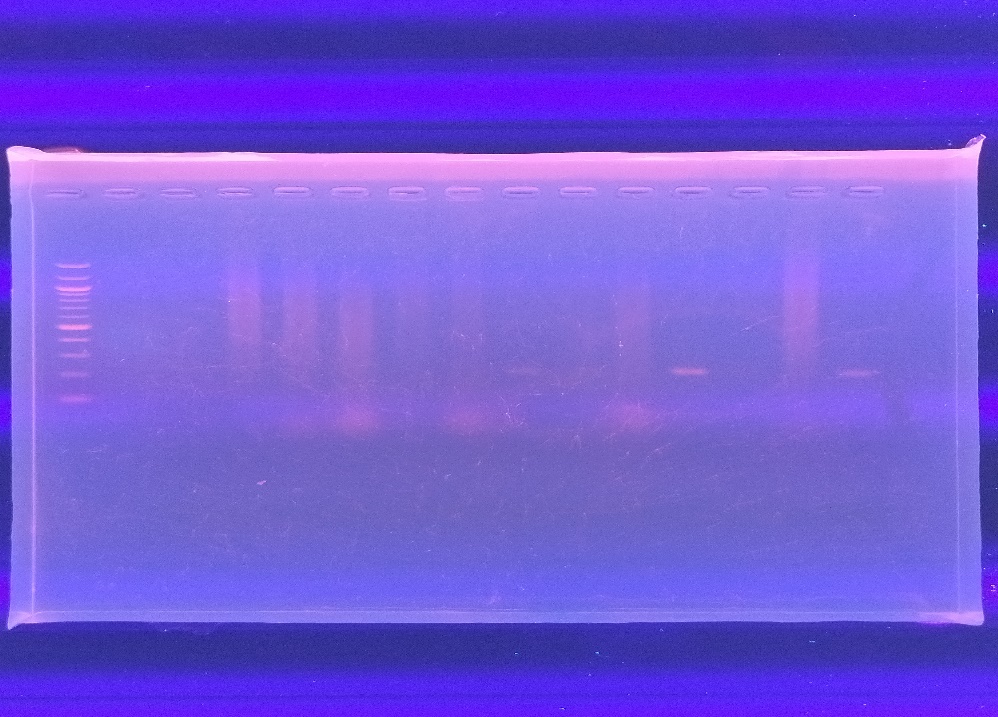


B

Figure 6: (A & B) are Amplified PCR products of *Erm C* at (190 bp). Lane M: 100 bp ladder lane. Lane 1, 2, 22, 25 and 28 positive to *Erm C* gene for Erythromycin resistant *S. aureus*.
